# Clinical Reports of Cases in the Medical Wards of Mercy Hospital

**Published:** 1872-10-01

**Authors:** N. S. Davis


### Clinical Reports.

### CLINICAL REPORTS OF CASES IN THE MEDICAL WARDS OF MERCY HOSPITAL.

SERVICE OF PROF. N. S. DAVIS.

CASE I. *Cirrhosis and Albumina in the same Patient.*—This patient is a laboring man, formerly a volunteer soldier, aged about 30 years; he was brought into the hospital about five weeks since. He claimed to have been sick only four or five weeks previous to his admission. At that date his countenance was pale and expressive of anxiety; his extremities cool and a little livid; pulse small, weak, and quick; respiration short and hurried; mouth and tongue moist; bowels quiet; urine scanty and high colored; and some dull pain in the back and loins. The feet, legs, and all the dependent parts of the body, were swollen from dropsical or serous infiltration into the subcutaneous areolar tissues. The peritoneal cavity was also so largely distended with serous effusion as to press on the diaphragm and much impede respiration. His abdomen showed marks of two recent tappings, and he stated that he had been tapped three times in as many weeks, the last time only three days before he was brought to the hospital.

The general prostration of the patient, the feebleness of circulation, the extent of the dropsical effusions, and the rapidity of the filling up of the peritoneal cavity after the tappings, appeared to indicate an unfavorable result. So far as could be learned, the symptoms had supervened gradually without any indications of peritonitis or other acute inflammation. The urine, tested by heat and nitric acid, showed a large coagulation of albumen, which would explain the general anasarca, but not the rapid accumulation in the abdominal cavity. This latter plainly indicated some local obstruction to the portal circulation. The results of careful percussion and palpation were such as to make it probable that the liver was smaller than natural, or cirrhased.

The co-existence of albuminuria and cirrhosis in the same patient is not of frequent occurrence, yet it is quite evident that we have both in the case before us. The present indications for treatment were, to counteract the increase of dropsical effusions by increasing the action of the skin and kidneys, and to sustain the strength and nutrition of the patient. For these purposes he was directed the two following prescriptions:

℞ Tinc. Digitalis, ℥ i

Fl. Ext. Lactuea, ℥ ij

Syrup. Prums Virg., ℥ i

Iodide Potass., ℥ iij

Mix. Take one teaspoonful every six hours.

℞ Spts. Nit. Dulc., ℥ iij

Tinc. Ferri Chlod., ℥ i

Mix. Take one teaspoonful every six hours, alternating it with the other medicine.

He was directed milk, beef tea, and other easily digested food, but no stimulating drinks. As the lower extremities and scrotum, as well as the cavity of the abdomen, were already tense with the dropsical effusion a free incision was made into each ankle to allow the serous fluid to escape. Under this treatment the general anasarca was almost entirely relieved; the extremities became warmer and better color; the pulse more full and less frequent, and his appetite and digestion good. Still the effusion into the peritoneal cavity slowly increased, and three weeks after his admission he became so distressed from interference with respiration that he was tapped and about four gallons of fluid of a milky white color drawn from the abdominal cavity. After the removal of the fluid, a careful examination of the right hypochondrium confirmed the evidences of atrophy or cirrhosis of the liver.

The same internal treatment was continued as before stated, and the patient has continued to improve slowly in his general health and nutrition, but the ascites has returned to such an extent that now, only two weeks after the previous tapping, the abdomen is seen largely distended, tense, and fluctuating on percussion. The respiration is again embarrassed, and the patient urgently desired to have the tapping repeated. This was done by the professor in the presence of the class, and between three and four gallons of water, or whitish serum, drawn off. It was explained that the tapping was a measure of temporary relief only, and that if the diagnosis in reference to the existence of albuminuria and cirrhosis was correct, the prognosis must be positively unfavorable. The tonics, diuretics, diaphoretics, and nourishment, with occasional tapping, might render the patient more comfortable and somewhat prolong life, but could not remove the pathological changes in the kidneys and liver.

Case II. *Paraplegia.*—This patient is a man about 40 years of age; native of Ireland; and has been in the hospital two or three months. He is thin in flesh; pale or anæmic; his eyelids affected with chronic inflammation; tongue clean; respiration and pulse natural; skin rather cool; appetite and digestion moderate; bowels nearly regular once a day; and urine also nearly natural in quantity. But the patient has only partial control over the passage of either urine or feces, and can exert only a very limited motor influence over the lower extremities; merely being able to draw up the feet a little and extend them again, when lying down. The lower extremities are also extremely emaciated and nearly destitute of sensibility, yet subject to frequent spasmodic twitchings.

At the time the patient was first admitted to the hospital, all the parts below the crest of the ilium and the junction of the lumbar vertebra with the sacrum, were completely paralyzed, both in regard to sensation and motion. The feces and urine passed without the knowledge or control of the patient, and the lower extremities were entirely motionless and anæsthetic.

He is a man who has long been addicted to the use of intoxicating drinks, and had been under treatment by some physician for the chronic affection of his eyelids some time before he was attacked with the paraplegia. It was remarked by the lecturer that this case was undoubtedly one arising primarily from morbid nutrition of the lower third of the spinal cord. This class of patients keep up the use of such drinks as interfere with the natural metamorphose of tissue and excretion, especially of the waste carbonaceous matter, and thereby encourage fatty degenerations of structure in various parts of the body. In most cases these structural alterations take place most prominently in the liver and kidneys, as in Case I, just described; but in others the change appears to affect the coats of the vessels in the brain and spinal cord, weakening them by the deposit of fat granules, until in the brain they yield to the pressure of the contained blood permitting extravasation and apoplexy, or hemiplegia. The same process in the spinal cord would result in the production of various morbid sensations and weakness of the lower extremities, and when the extravasation takes place, more or less complete paraplegia. The prognosis in such cases was stated to be decidedly unfavorable. Under the influence of proper nourishment, good air, and the judicious use of tonics (not exhilarants), they will often slowly improve, until, as in this case, they regain some sensibility and a very limited power of motion in the paralyzed parts. At that point the improvement usually ceases, and the patients remain apparently almost stationary for weeks and months; but in truth the nervous centers involved are undergoing atrophy, or softening from defective nutrition, and eventually the patients die from asthenia.

This patient has been taking a combination of citrate of iron, quinine, and extract of nux vomica during most of the time that he has been in the hospital. When the spasmodic twitchings have been troublesome and his nights restless, he has had from 15 to 20 grs. of either bromide of potass. or of hydrate of chloral at bedtime. His diet has been plain and nutritious; and constant attention given to cleanliness and proper ventilation. Close attention to the hygienic management of such patients is of the highest importance; otherwise their imperfect control over their evacuations will keep the bed foul, the air of the room impure, and speedily induce large and tormenting sloughs or bed sores on the hips and sacrum, and materially hasten the final exhaustion of the patient.

